# The Protocol for the Multi-Ethnic, multi-centre raNdomised controlled trial of a low-energy Diet for improving functional status in heart failure with Preserved ejection fraction (AMEND Preserved)

**DOI:** 10.1136/bmjopen-2024-094722

**Published:** 2025-01-28

**Authors:** Joanna M Bilak, Iain Squire, Joanne V Wormleighton, Rachel L Brown, Michelle Hadjiconstantinou, Noelle Robertson, Melanie J Davies, Thomas Yates, Mehak Asad, Eylem Levelt, Jiliu Pan, Oliver Rider, Fardad Soltani, Christopher Miller, Gaurav Singh Gulsin, Emer M Brady, Gerry P McCann

**Affiliations:** 1 Department of Cardiovascular Sciences, University of Leicester and the National Institute for Health Research Leicester Biomedical Research Centre, Glenfield Hospital, Leicester LE3 9QP, UK; 2 Department of Neuroscience, Psychology and Behaviour, University of Leicester, University Road, Leicester LE1 7RH, UK; 3 Leicester Diabetes Research Centre, Leicester General Hospital, Gwendolen Road, Leicester LE5 4PW, UK; 4 Multidisciplinary Cardiovascular Research Centre and Biomedical Imaging Science Department, Leeds Institute of Cardiovascular and Metabolic Medicine, University of Leeds, Leeds LS2 9JT, UK; 5 Oxford Centre for Clinical Magnetic Resonance Research, University of Oxford, John Radcliffe Hospital, Headley Way, Oxford OX3 9DU, UK; 6 Division of Cardiovascular Sciences, School of Medical Sciences, Faculty of Biology Medicine and Health, University of Manchester, Manchester Academic Health Science Centre, Oxford Road, Manchester M13 9PL, UK; 7 BHF Manchester Centre for Heart and Lung Magnetic Resonance Research, Manchester Academic Health Science Centre, Manchester University NHS Foundation Trust, Manchester Academic Health Science Centre, Southmore Road, Manchester M13 9LT, UK

**Keywords:** Heart failure, Obesity, Diabetes Mellitus, Type 2, Cardiovascular Disease, Magnetic Resonance Imaging, Quality of Life

## Abstract

**Introduction:**

Heart failure with preserved ejection fraction (HFpEF) is characterised by severe exercise intolerance, particularly in those living with obesity. Low-energy meal-replacement plans (MRPs) have shown significant weight loss and potential cardiac remodelling benefits. This pragmatic randomised trial aims to evaluate the efficacy of MRP-directed weight loss on exercise intolerance, symptoms, quality of life and cardiovascular remodelling in a multiethnic cohort with obesity and HFpEF.

**Methods and analysis:**

Prospective multicentre, open-label, blinded endpoint randomised controlled trial comparing low-energy MRP with guideline-driven care plus health coaching. Participants (n=110, age ≥18 years) with HFpEF and clinical stability for at least 3 months will be randomised to receive either MRP (810 kcal/day) or guideline-driven care for 12 weeks. Randomisation is stratified by sex, ethnicity, and baseline Sodium Glucose Cotransporter-2 inhibitor (SGLT2-i) use, using the electronic database RedCap with allocation concealment. Key exclusion criteria include severe valvular, lung or renal disease, infiltrative cardiomyopathies, symptomatic biliary disease or history of an eating disorder. Participants will undergo glycometabolic profiling, echocardiography, MRI for cardiovascular structure and function, body composition analysis (including visceral and subcutaneous adiposity quantification), Kansas City Cardiomyopathy Questionnaire (KCCQ) and Six-Minute Walk Test (6MWT), at baseline and 12 weeks. An optional 24-week assessment will include non-contrast CMR, 6MWT, KCCQ score. Optional substudies include a qualitative study assessing participants’ experiences and barriers to adopting MRP, and skeletal muscle imaging and cardiac energetics using 31Phosphorus MR spectroscopy.

**Statistical analysis:**

Complete case analysis will be conducted with adjustment for baseline randomisation factors including sex, ethnicity and baseline SGLT2-i use. The primary outcome is the change in distance walked during the 6MWT. The primary imaging endpoint is the change in left atrial volume indexed to height on cardiac MRI. Key secondary endpoints include symptoms and quality of life measured by the KCCQ score.

**Ethics and dissemination:**

The Health Research Authority Ethics Committee (REC reference 22/EM/0215) has approved the study. The findings of this study will be published in peer-reviewed journals.

**Trial registration number:**

NCT05887271.

**STRENGTHS AND LIMITATIONS OF THIS STUDY**

- This is a pragmatic, randomised controlled trial exploring the efficacy of a low-energy meal-replacement plan (MRP) on exercise intolerance.
- Primary endpoint is distance covered in a Six-Minute Walk Test, chosen in collaboration with people living with heart failure with preserved ejection fraction (HFpEF), given it is most reflective of exercise intolerance which is the key daily limiting factor of HFpEF.
- Addition of comprehensive imaging (echocardiography, skeletal muscle and cardiac MRI) will allow the assessment of potential mechanisms of benefit.
- The nested qualitative study will allow for exploration of the participants’ barriers and facilitators to long-term adoption of MRP and the implementation of lifestyle changes.
- Limitations include the relatively small sample size, aiming for proof of efficacy and the small number of centres may limit the generalisability of the findings to the wider HFpEF population living with obesity.

## Background

 Heart failure with preserved ejection fraction (HFpEF) is increasingly prevalent and associated with high symptom burden and significant functional impairment.^1 2^ Over 80% of patients with HFpEF are either overweight or obese^3^ and emerging evidence suggests that excess body fat plays a direct role in the development and progression of HFpEF.^4^,^6^ Excess visceral adiposity promotes inflammation, hypertension, insulin resistance and dyslipidaemia resulting in increased risk of cardiovascular (CV) events. It is also associated with adverse cardiac remodelling,^7 8^ reductions in myocardial perfusion reserve (MPR)^9^ and myocardial energetics,^10^ all of which are implicated in the pathogenesis of HFpEF. Furthermore, obesity predisposes to skeletal muscle dysfunction, sarcopenia and frailty which exacerbate exercise intolerance, the key symptom of HFpEF.^11^,^17^ In recognition of the pathophysiological importance that comorbidities play in the genesis of HFpEF, the recent heart failure guidelines advocate the treatment of obesity and other systemic diseases as key therapeutic targets in HFpEF.^18^

Recent evidence from the semaglutide in obesity-related HFpEF (STEP-HFpEF)^19^ and semaglutide in obesity and type 2 diabetes mellitus-related HFpEF (STEP-HFpEF DM)^20^ trials highlights weight loss as a promising therapeutic target for improving exercise tolerance and reducing symptoms in heart failure patients. The STEP-HFpEF trial demonstrated that weekly 2.4 mg semaglutide injections resulted in 10.6% greater weight loss and a 20.3 m improvement in the Six-Minute Walk Test (6MWT) (95% CI 8.6 to 32.1; p<0.001) compared with placebo.^19^ Quality of life significantly improved, with a mean KCCQ score change of 7.8 points (95% CI 4.8 to 10.9; p<0.001).^19^ Similar benefits were observed in the STEP-HFpEF DM trial,^20^ where semaglutide led to larger reductions in heart failure symptoms (mean KCCQ score difference of 7.3 points, 95% CI 4.1 to 10.4; p<0.001), a 14.3 m improvement in the 6MWT (95% CI 3.7 to 24.9; p=0.008) and greater weight loss (difference of −6.4 percentage points; 95% CI −7.6 to −5.2; p<0.001) at 1 year compared with placebo.^20^

Substantial weight loss comparable to pharmacotherapy can be achieved through lifestyle interventions. In the largest lifestyle study to date, a 2×2 factorial randomised controlled trial of 100 HFpEF patients with obesity was randomised into the diet (400 kcal/day deficit), exercise (1-hour thrice-weekly supervised exercise session), or a combination of both for 20 weeks.^21^ The small average weight loss of 7 kg in the dietary intervention was associated with a 7-point improvement in the KCCQ score (95% CI (2.6 to 12.3), p=0.004), small but significant rise in peak VO_2_ by 1.3 (0.8, 1.8) mL/kg body mass/min and a 26 m increase in 6MWT distance (difference (95% CI) 25.9 (11.9 to 40.2) m, p<0.001). Additionally, a non-significant numerical decrease in left ventricular (LV) mass and LV relative wall thickness and a trend in improvement in diastolic function was shown by echocardiography.^21^

Although evidence from randomised controlled trials indicates that weight loss improves exercise tolerance and reduces symptoms in obesity-related HFpEF, existing trials primarily involved Caucasian and male participants (50% females in STEP programme), limiting their generalisability. This is particularly relevant as non-white ethnicities and females are at risk of higher symptom burden from HFpEF, hospitalisations and adverse outcomes.^22^,^24^ Semaglutide, while effective, has side effects (between 13% and 18% of users experienced adverse events)^19 20^ and may not be suitable for all patients or desirable by those with polypharmacy or needle aversion. The dietary intervention in Kitzman *et al*’s study produced modest weight loss but was impractical, requiring individually prepared meals and the trial was conducted in an inpatient setting.^21^ Furthermore, the trials used basic CV phenotyping,^19^,^21^ leaving the effects on key HFpEF indices, such as left atrial and ventricular remodelling, aortic distensibility, MRP and cardiac fibrosis, unknown. In particular, changes in left atrial volume indexed (LAVi) have been shown to be of prognostic significance in HFpEF.^25^ Thus, there remain uncertainties relating to the mechanisms of benefit of weight loss on CV outcomes and the need for further study on the direct impact on CV remodelling. The impact on frailty, sarcopenia or body composition changes, and how those related to cardiac remodelling or exercise tolerance also remains unclear.

### Aims

The aim of this study is to assess the efficacy of an meal-replacement plan (MRP) in improving exercise intolerance, symptoms, quality of life, CV remodelling and skeletal myopathy in patients living with HFpEF and obesity.

The nested qualitative substudy aims to enhance our understanding of the attitudes and barriers to the adoption of MRP (see online supplemental appendix 3 for the interview topic guide) The musculoskeletal substudy will elicit the effects of weight loss on sarcopenia, muscle strength, composition and changes in skeletal muscle energetics in response to weight loss. At selected sites, the optional 31-Phosphorus cardiac MR spectroscopy (31P-MRS) to assess changes in skeletal muscle and myocardial energetics in response to weight loss.

The optional 24-week visit aims to provide data on delayed cardiac remodelling, and remote follow-up at 12 months will provide outcome data related to mortality and hospitalisations.

## Methods and analysis

### Trial design

The Mult-Ethnic, multi-centre raNdomised contolled trial of a low energy Diet for impriving functional status in heart failure with Preserved ejection fraction (AMEND-preserved) is an open-label, multicentre, randomised controlled trial conducted at secondary care hospitals in the UK in Leicester, Manchester, Leeds and Oxford. The trial is registered on ClinicalTrials.gov NCT05887271 with approval from the national research ethics service (22/EM/0215).

**Figure 1:**
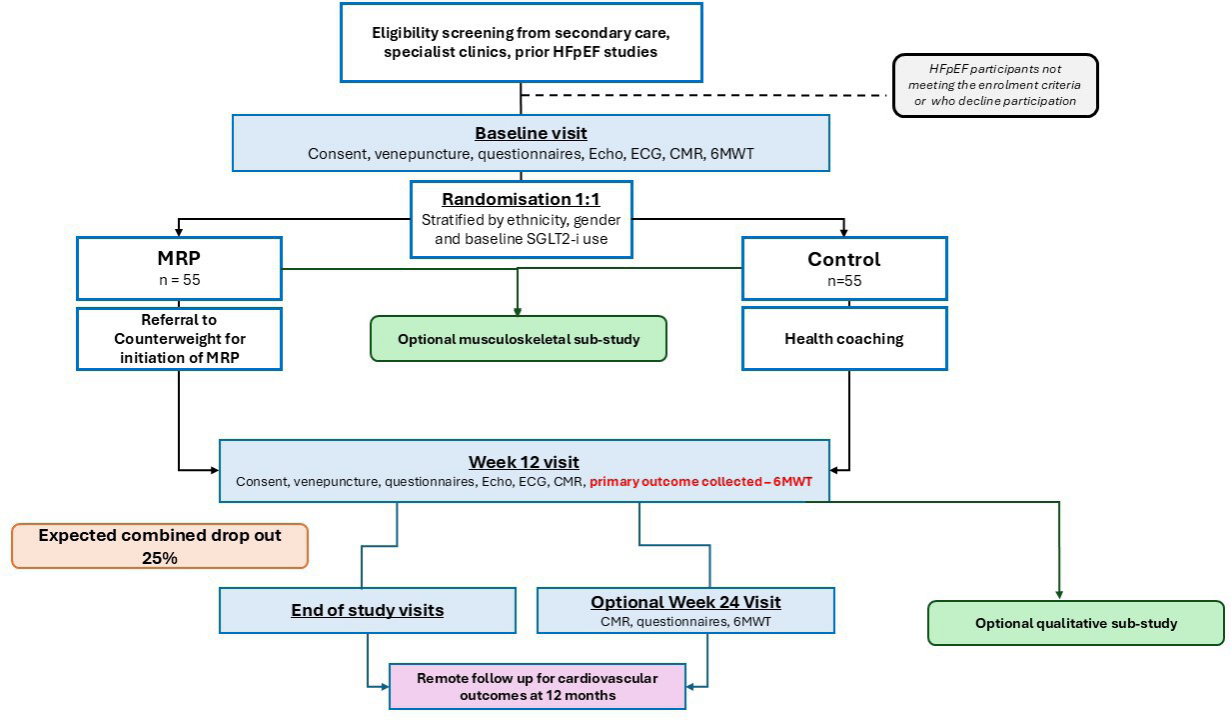
Summary of trial flow. Participants in the Multi-Ethnic, multi-centre raNdomised, controlled trial of a low-energy Diet for improving functional status in Heart failure with Preserved ejection fraction (AMEND Preserved) trial will be recruited from secondary care and randomised 1:1 into meal replacement plan (MRP) or standard care (control) for a duration of 12 weeks. Primary outcome (Six-Minute Walk Test, 6MWT) will be collected at baseline and 12 weeks. Participants will be invited to participate in the optional qualitative interview and sarcopenia substudies, as well as offering an optional visit at week 24 to assess for delayed cardiac remodelling. Health outcomes will be collected remotely at 12 months. HFpEF, heart failure with preserved ejection fraction. CMR: cardiac magnetic resonance imaging, ECG: electrocardiogram

Potential participants will be identified from heart failure clinics, hospital wards, diagnostic departments, health records and waiting lists as detailed in figure 1. To enhance study accessibility and include under-represented groups, recruitment will extend to community settings such as faith centres and community groups, aiming to achieve a balanced ethnic mix. We collaborate with the Leicester Centre for Ethnic Health Research for guidance on broadening participation. Our recruitment strategies will be constantly monitored to ensure a wide representation of ethnicities and genders. All participants meeting the eligibility criteria (summarised in box 1) will be invited to participate in the study.

Box 1 **Eligibility criteria for inclusion in the AMEND Preserved trial**

**Inclusion criteria**

- Established clinical diagnosis of heart failure with preserved ejection fraction (HFpEF) (left ventricular ejection fraction >45%) made by a cardiologist or a primary care physician with heart failure expertise, or a heart failure specialist nurse.
- Clinically stable for ≥3 months (no admissions to hospital).
- Obesity (body mass index ≥30 kg/m^2^ if white European or ≥27 kg/m^2^ if Asian, Middle Eastern or black ethnicity).
- Age ≥18 years old.

**Exclusion criteria**

- Inability to walk/undertake Six-Minute Walk Test.
- Inability to follow a low-energy meal replacement plan.
- HFpEF due to infiltrative cardiomyopathy (cardiac amyloidosis or sarcoidosis), genetic hypertrophic cardiomyopathy, restrictive cardiomyopathy/pericardial disease or congenital heart disease.
- Known heritable, idiopathic or drug-induced pulmonary arterial hypertension.
- Severe chronic obstructive pulmonary disease (forced expiratory volume in 1 s<1.0 L).
- Severe primary valvular heart disease.
- Anaemia (haemoglobin<100 g/L).
- Severe renal disease (estimated glomerular filtration rate <30 mL/min/1.73 m^2^).
- Unintended weight loss >5 kg in preceding 3 months.
- Symptomatic gallstone disease including biliary colic or recent cholecystitis (<3 months).
- Active substance abuse (drugs or alcohol).
- History of bariatric surgery in the last 3 years.
- Active illness likely to cause change in weight.
- Women who are pregnant or are considering pregnancy.
- People currently participating in another clinical research trial that is likely to affect diet or weight change.
- History of a severe mental illness including an eating disorder.
- Individuals with a diagnosis of type 1 diabetes mellitus.

### Study visits

The study will comprise two main assessment visits, at baseline and 12 weeks, with an optional third visit at week 24. An outline of study visits and measures taken during each visit is shown in table 1. Participants will have the option to split the baseline and 12-week visits into two visits in close succession if undergoing the assessments in 1 day is too onerous. Participants will be offered reimbursement for travel. Lunch will be provided during each assessment visit.

**Table 1:** Overview of assessments during study visits

	Baseline visit	Week 12 visit	Week 24 visit (optional)
Visit window		±3 days	±2 weeks
Eligibility assessment	✓		
Consent	✓		
Medical history and examination	✓		
Medication review and adjustment	✓	✓	
Demographics and height measurement	✓		
Body weight recording	✓	✓	✓
Blood pressure and heart rate	✓	✓	✓
Blood sampling	✓	✓	
ECG	✓	✓	
Echocardiography	✓	✓	
CMR	✓	✓	✓
Accelerometery	✓	✓	
Health coaching (in both arms at baseline then control arm only)	✓	✓	
6MWT	✓	✓	✓
Handgrip strength	✓	✓	
Questionnaires	✓	✓	✓

6MWTSix-Minute Walk Test

### Baseline visit

Following eligibility confirmation by the study clinician and after obtaining written informed consent (an example consent form is provided in online supplemental appendix), all participants will undergo a clinical review and medication adjustment with the study clinician(s) in line with current clinical guidance for HFpEF.^26^,^28^ All participants will proceed to have a comprehensive multiparametric phenotyping (assessments outlined below). All participant data will be anonymised and stored in a secure electronic database.

### 12-week data collection visit

At 12-week visit, baseline assessments as described below will be repeated alongside a comprehensive health review. Medications paused at the trial’s onset will be reassessed and resumed if necessary. Primary outcome will be collected on this visit; however, participants will be invited to take part in the optional substudies and additional visit at 24 weeks as detailed below.

Clinical records will be followed up and reviewed at 12 months for clinical outcomes.

### Data collection and management

The study team will input all data, with accuracy verified by the principal investigators (PIs). Data quality control will involve identifying missing data, outliers and discrepancies through systematic queries. Each participant will receive a unique study ID on enrolment. Anonymised participant data will be securely uploaded and managed using the Research Electronic Data Capture (REDCap) platform.

### Assessments at baseline and week 12 visits

The following assessments will be conducted in a single or split visit depending on the participant’s preference. The participants will attend after an overnight fast and a light meal will be provided postvenepuncture.

#### Anthropometric variables

Body weight and height will be measured to the nearest 0.1 kg and 1 cm, respectively. Brachial blood pressure (BP) will be obtained from the participant’s dominant arm in a seated position and an average of the last two measurements will be recorded. Ethnicity, smoking status and weekly alcohol intake, medical and medication history will be obtained by self-report and from clinical records where applicable.

#### Biochemical variables

Fasting blood sample will be taken for biochemical profile for diabetes screening/glycaemic control in those with known type 2 diabetes (T2D) (fasting glucose and glycated haemoglobin (HbA1c), respectively), liver and kidney function, lipid profile (including total cholesterol, high-density lipoprotein (HDL), non-HDL cholesterol and triglyceride content), assessment of blood markers of inflammation (C reactive protein, CRP) and measures of cardiac stress (high sensitivity-troponin and N terminal brain-natriuretic peptide levels, NT-proBNP). Additional plasma and serum will be stored for future metabolomic, proteomic and genomics analyses subject to additional funding.

#### Questionnaires

The KCCQ^29^ will be used to assess disease-specific quality of life. All participants in the study will also undergo simple assessment of frailty and sarcopenia including the Edmonton Frail Scale (EFS) questionnaire, which provides an objective measure of frailty and the impact on physical, cognitive, social and general health domains.^30^ The ‘Strength, Assistance with walking, Rise from a chair, Climb stairs and Falls’ (SARC-F) questionnaire assesses strength, mobility and falls risk.^31^

#### Skeletal muscle assessment and strength testing

Participants’ quadriceps muscle volume will be quantified through MRI, complemented by handgrip strength testing via a dynamometer, with participants seated, elbow resting at a 90° angle, performing maximal effort squeezes.^32^ All three readings will be recorded but the highest is taken as a key measure. These baseline assessments of frailty and sarcopenia are separate but complementary to the optional musculoskeletal substudy discussed later in the manuscript.

#### Six-Minute Walk Test

Functional exercise capacity will be assessed using a 6MWT according to established guidelines in all participants.^33^ Participants will be advised to wear comfortable clothing and shoes prior to attending their visit. The 6MWT will be performed by a trained clinical member of research team blinded to treatment allocation. Participants will be allowed rest for 10 min prior to the 6MWT, conducted in a 6 m hallway with 3 m interval cones, facilitating back-and-forth walking at a self-determined pace and the same protocol will be employed on all recruitment centres. The distance traversed is determined by the number of completed laps multiplied by the corridor length. Dyspnoea will be assessed using Borg’s scale at the start and finish.

#### Accelerometery

A 7-day accelerometery will be performed at baseline and 12 weeks to assess change in daily physical activity and sedentary time. Participants will be required to wear an accelerometer watch on their non-dominant wrist for seven consecutive days and keep a daily log of their sleep and wake times, including periods when the accelerometer is removed. After 7 days, they will return the log and accelerometer in a prepaid envelope. Acceleration data, captured at 100 Hz, will be analysed using commercially available software.

#### Transthoracic echocardiography

All participants will undergo transthoracic echocardiography performed by British Society of Echocardiography accredited physiologists at baseline and week 12 visits.^34^ Assessment of tissue Doppler indices of diastolic filling and speckle tracking for global longitudinal strain, LV size and systolic function and valve function will be undertaken.^34^

#### MRI

**Figure 2:**
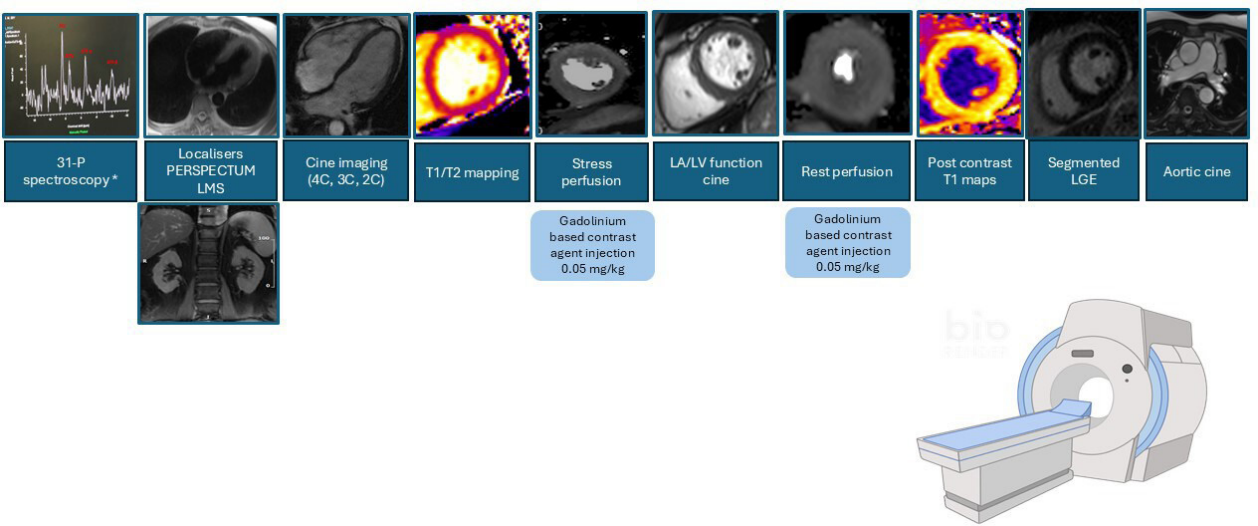
Cardiac Magnetic Resonance (CMR) imaging protocol. CMR imaging will be undertaken at 1.5 or 3-Tesla with optional 31-phosphorus spectroscopy at centres where available. Baseline and week 12 CMR will be performed at the same field strengths. 2C/3C/4C: two chambers, three chambers, four chambers.LA: left atrium, LGE: late gadolinium enhancement; LMS: liver multi scan; LV: left ventricle

Unless contraindicated, all participants will be invited to undergo cardiac MRI (CMR) with assessment of body composition and organs (including liver, kidneys and pancreas), as outlined in figure 2. Participants who are unable to tolerate the full protocol will be offered a shortened ‘core dataset’ CMR protocol, discussed later in the text. MRI will be performed using either a 1.5 or 3-Tesla platform with an 18-channel phased-array cardiac receiver coil.^35^ Baseline and follow-up scans will be completed at the same field strength.

#### MRI assessment of body composition

Each participant’s body composition, liver, pancreas and kidneys will be assessed using a multiparametric imaging tool COVERSCAN (PERSPECTUM, Oxford, UK) which will be conducted before the cardiac sequences are acquired. The COVERSCAN has been validated in large prospective studies of multiorgan effects of COVID-19 with methodology previously published.^36^,^39^ To assess body composition, a stack of 3–4 axial 3D T-1 weighted multiecho gradient echo Dixon VIBE sequence will be acquired extending from the top of ninth thoracic vertebrae (T9) to the tip of the femoral condyle.

Liver and pancreas multiparametric assessment will be conducted using the LiverMultiScan (LMS) acquisition protocol (PERSPECTUM, Oxford, UK)^40^ with assessment of liver adiposity (with proton density fat fraction (PDFF)), liver iron content (using T2*) and inflammation (with T1 Modified Look-Locker Inversion recovery pulse sequence (MOLLI)).^40^ For the assessment of pancreas, a PDFF map and an MOLLI T1 map will be acquired showing the head body and tail of pancreas in a single slice.

A single coronal T1 map of both kidneys will also be acquired.

#### Cardiac Magnetic Resonance assessment of myocardial structure, function and perfusion

Following localisers, balanced steady-state free precession (bSSFP) LV cine images will be acquired in two-chamber, three-chamber and four-chamber views. Myocardial tissue will be characterised with a single T1 map (MOLLI) acquired at the junction of the basal/mid LV short-axis level and a single T2 map at junction of the basal/mid LV short-axis level acquired pre-contrast. Unless contraindicated, participants will undergo stress and rest perfusion with pharmacological vasodilator adenosine (140 µg/kg/min for 2–3 min). If a satisfactory haemodynamic response is not achieved (a drop in systolic BP of 10 mm Hg and/or a rise in heart rate of 10 bpm) after 2 min, the dose will be increased to a maximum of 210 µg/kg/min. During peak stress, a gadolinium-based contrast agent (gadoterate meglumine or gadobutrol, depending on availability at each recruitment site) will be injected (at 0.05 mmol/kg) at 5 mL/s, followed by a 20 mL bolus of normal saline. Data will be acquired using a multislice, free breathing saturation recovery pulse sequence with fast low-angle short readout acquired over 60 heart beats, in three short-axis slices as previously described.^41^ Myocardial blood flow (MBF) will be quantified using a dual pulse sequence acquisition with fully automatic inline calculations as previously described.^8 42^ Rest images will be performed approximately 10 min after stress with a further 0.05 mmol/kg bolus of contrast. In the meantime, short-axis stack images will be acquired using cine imaging (bSSFP) covering the entire LV. Late gadolinium-enhanced images (inversion recovery gradient echo sequence) and postcontrast T1 maps (MOLLI) will determine focal and diffuse myocardial scar, respectively. Aortic cine images will be acquired in the ascending and descending aorta at the level of the pulmonary artery bifurcation. Pulse pressure will be measured simultaneously to allow calculation of aortic distensibility which has been postulated to be the key determinant of LV remodelling in T2D.^43^

#### The ‘core data set’ CMR protocol

Core dataset CMR protocol will be employed at the supervising clinician’s discretion for patients unable to lie flat for the duration of the full protocol. To assess body composition, a stack of 3–4 axial 3D T-1 weighted multiecho gradient echo Dixon VIBE sequence will be acquired extending from the top of ninth thoracic vertebrae (T9) to the tip of the femoral condyle. This will be followed by localisers, a set of two-chamber, three-chamber and four-chamber cines, precontrast mid-level T1 map, an adenosine stress perfusion protocol, short-axis LV stack, postcontrast mid-level T1 map and a segmented LGE imaging, using the protocol as discussed above.

### Image analysis

The images will be anonymised and labelled with a unique study ID. Analysis of body composition, liver, pancreas and kidney imaging, as well as volumetric analysis of the left and right ventricular and atrial data, will be undertaken by PERSPECTUM.^40^ The quantitative assessment of myocardial perfusion, myocardial strain and strain rates will be performed by the trained core lab staff at Leicester blinded to all participant details and allocation groups.

#### Analysis of cardiac sequences

Cardiac volumes and mass, left and right ventricular stroke volume and ejection fraction will be calculated by PERSPECTUM.^37^ Experienced CMR analysts blinded to treatment allocation will use CVi42 (Cardiovascular Imaging, Canada) to manually trace the endocardial borders in the end-diastolic and end-systolic phases in each of the short-axis views, following the standard UK Biobank evaluation approach.^44^ This analysis will produce, for both the left and the right ventricle the end diastolic volume, end systolic volume, stroke volume and ejection fraction. Additionally, LV muscle mass and wall thickness will be determined from the function data.

Analysis of LV strain, quantitative analysis of perfusion data, calculation of the extracellular volume (ECV) will be performed by an experienced operator in Leicester using CVI42 software. Assessment of peak diastolic filling rate, systolic global longitudinal and circumferential strain, global longitudinal strain and circumferential and longitudinal peak early diastolic strain rates will be performed as previously described.^35^ Epicardial adipose tissue volume will be quantified in cubic centimetres.

Late gadolinium enhancement will be qualitatively assessed in all 16 segments of the American Heart Association model by an expert CMR cardiologist as per the Society for Cardiac Magnetic Resonance guidance.^45^ For ECV calculation, a single region of interest will be manually drawn in the LV septum of the short axis on the native and postcontrast T1 maps, as previously described.^46^ Segments containing myocardial infarction or non-ischaemic enhancement (as judged by an experienced CMR expert) will be excluded.^47^ ECV value will be calculated using the previously described formula.^46^

Quantitative analysis of perfusion data will be performed as previously described using an automated AI tool.^41^ The myocardial perfusion maps will be visually inspected for quality and discarded if errors are found. The MBF values for each of the 16 myocardial segments will be recorded for stress and rest, and global values will be automatically calculated as averages. Rate pressure product (RPP) will be calculated (heart rate×systolic BP) and rest MBF will be corrected for RPP as per best practice.^48^ Myocardial perfusion reserve (MPR) will then be automatically calculated for each segment as stress MBF/rest MBF and results recorded.^41^

Ascending, descending and mean aortic distensibility will be measured using Java Image Manipulation (Xinapse Software, Essex, UK), as previously described.^43^

#### Body composition and analysis of body composition and extracardiac organ images

For delineation of subcutaneous adipose tissue (SAT), visceral adipose tissue (VAT) and Skeletal Muscle Index (SMI), a single two-dimensional section positioned at the third lumbar (L3) vertebrae will be extracted from whole-body Dixon MRI. The L3 section will be selected as it has been shown to be the most representative of the body skeletal muscle distribution and it is accurate in the assessment of the total subcutaneous and visceral fat volumes.^49^,^51^ The cross-sectional areas of SAT, VAT and skeletal muscle will be manually segmented in the ITK-SNAP software (V.3.8.0) tool^50^ and the resulting values will be reported as centimetres squared. The SMI will be calculated by indexing the centimetres squared values of lean muscle to the squared height of the participant (centimetres squared/metres squared).^40^

Liver MRI scans will be analysed using PERSPECTUM’s LMS technology.^36^ This software automatically delineates the liver from cT1, T2* and PDFF image maps, excluding major vessels within the image section as previously described.^36^ For each liver, a metric of PDFF, T2* and cT1 (cT1 is a measurement of T1 that has been corrected for the confounding effects of iron and standardised to 3-Tesla) will be yielded as previously described.^36^ Pancreas images will be analysed by manually placing, on PDFF maps when possible, a single region of interest of 10 mm within the head, body, and tail of the pancreas, avoiding blood vessels and ducts. The output T1 will be standardised to 3 Tesla for both the liver and pancreas. Kidney T1 maps will be analysed by manually placing on T1 maps up to 10 regions of interest around the renal cortex.

### Optional assessments and substudies

#### Optional 24-week visit

Participants will be invited for an optional visit at 24 weeks, to assess the delayed remodelling with a focused non-contrast CMR scan to assess LV volumes, mass and aortic distensibility, a 6MWT and KCCQ score.

#### Optional qualitative substudy evaluation

Both MRP and control group participants will be invited for an optional focused semi-structured one-on-one interview after their 12-week visit to identify barriers and enablers to MRP, share their views on healthy eating and health, and their experiences. This includes those who complete the programme or drop out. Including participants from the control group will enable comparison of MRP experiences against health coaching, highlighting challenges in self-directed lifestyle changes. The interview topic schedule was created with input from a health psychologist and local patient and public involvement (PPI) group (available in online supplemental file: appendix 3).

#### Optional musculoskeletal substudy

At the Leicester site only, participants in the MRP and control group will be invited to take part in the musculoskeletal substudy with additional single visit for calf and quadriceps muscle strength testing, physical performance battery test,^52^ skeletal muscle biopsy^53^,^55^ and skeletal muscle ^31^P-MRS.^56^,^58^ Participation in this substudy is optional for participants.

#### ^31^Phosphorus MR spectroscopy

Where available at recruitment sites on a 3.0 Tesla MR system, participants will be offered the option of undergoing ^31^P-MRS at the beginning of the cardiac protocol, using methodology as described previously.^59^ In brief, ^31^P-MRS will be performed to assess myocardial PCr/ATP ratio from a voxel placed in the midventricular septum, with patients lying supine and a ^31^P-transmitter/receiver cardiac coil (Rapid Biomedical) placed over the heart on a 3.0 T MRI system.^59 60^

### Randomisation

Randomisation (1:1) will occur at the end of the baseline visit after data collection, using the ‘blockrand’ package in R^61^within the REDCap (Research Electronic Database Capture) system . The allocation list will be concealed from the user and non-editable once uploaded. REDCap’s randomisation module will reveal participant allocation only at the moment of randomisation, ensuring strict allocation concealment. The randomisation will be stratified by sex, ethnicity and baseline SGLT2-i use.

### Study interventions

#### Baseline optimisation of medical therapy for heart failure

All eligible participants entering the study will be offered a sodium glucose co-transporter 2 inhibitor (SGLT2-i), if not already prescribed, in line with the current National Institute for Care and Clinical Excellence guidance.^27^ There will be an approximate 4-week interval between the commencement of SGLT2-i and the start of MRP to take effect in order to observe its full metabolic and CV outcome impact.^27^ Given the evidence of benefit of SGLT2-i in HFpEF and the low risk of hypoglycaemia and hypotension is minimal,^32^ SGLT2-i will not be stopped during MRP. Individuals with underlying type 2 diabetes mellitus will be provided ketone measuring strips on initiation of MRP.

#### Meal replacement plan

Those allocated to the MRP will be referred to our commercial partner Counterweight and follow the ‘Counterweight Plus’ program with 12-week MRP.

The MRP follows ~810 kcal/day meal replacement diet with a macronutrient ratio of 40% protein, 50% carbohydrates and 10% fat. The MRP regimen includes four daily meal packs, 100 mL of semiskimmed or non-dairy milk, non-starchy vegetables such as leafy greens or cruciferous vegetables and encourages ~2 L of non-caloric fluid intake daily. The diet transitions to maintenance after achieving a 50% excess weight loss or by week 12. Those who are morbidly obese (body mass index ≥40 kg/m^2^) at baseline will undergo a stepwise gradual calorific reduction from approximately 1500 kcal/day to 810 kcal a day over 1–2 weeks, depending on baseline body weight.

Alongside the MRP, all participants receive health behaviour coaching and medication adjustments by clinicians, with biweekly clinical reviews and remote monitoring tools provided for safety which include automatic BP machine and weighing scales, and ketone strips in those with T2D. Medications will be reviewed prior to starting the diet by the study clinician(s) and will be advised to stop or reduce antihypertensives and glucose-lowering agents as deemed appropriate. The approach is outlined in online supplemental file: appendices 1 and 2 for adjustment protocols for antihypertensives and glucose-lowering medications. Glucose and BP monitoring will guide clinical reviews, conducted either in person or remotely based on participant preference.^62^ Routine reviews by Counterweight dieticians will occur remotely every 2 weeks. Based on participant and PPI feedback, the number of contacts with the research team has been minimised to baseline and follow-up visits only. However, in the event of participant’s request for a review or medical issues arising during the study, a review with the study clinician(s) will be arranged. MRP participants will use digital weighing scales and BP monitors for home monitoring. The use of liquid fibre (ispaghula husk) will be recommended to all participants on MRP to reduce risk of constipation. Individuals with coexisting T2D will be provided with ketone measuring strips and advised on sick day rules with respect to their continuation of SGLT2-i therapy while on MRP. Individuals with existing gallstones will be offered a prescription of ursodeoxycholic acid to reduce the risk of flare-ups.

The ‘Counterweight Plus’ program, provided by Counterweight includes dedicated personal support from a dedicated Counterweight registered research dietitian and will have regular 1:1 appointments throughout the programme and up to 12 months post-trial. The diet programme is delivered via a digital application (‘App’) that is downloaded onto a smart phone or tablet. The Counterweight App functions include logging measurements, for example, weight, mood, reviewing progress, nutrition/physical activity educational content and goal setting. Individuals will be allocated a named ‘Counterweight dietitian/coach’ for personal support, for regular appointments and to moderated online ‘chat’ facility to enable peer support between participants, as recommended by our patient and PPI group feedback. Counterweight dietitians have undertaken formal competency-based training from Counterweight specialist dietitians, then ongoing supervision and mentoring, to maintain programme fidelity.

### Cessation of total MRP and reintroduction of normal diet

Decision whether to stop the MRP will be made at week 8, if the participant has met the 50% excess weight loss target. If this target is not met, the participant will continue with MRP for a further 4 weeks to a total of 12 weeks.

### Maintenance of weight loss

- Food reintroduction/maintenance phase (week 12 to month 12)
  - Stepped food reintroduction or transition period (eg, carbohydrate and fat reintroduction for the low carb and low fat diet options) will be individualised to participants and delivered by Counterweight dieticians.
  - Standard evidence-based behaviour change techniques including self-monitoring (of behaviours and behavioural outcomes), goal-setting and self-rewards, action-planning, problem-solving will be employed.
  - Digital delivery with monthly dietitian/coach support, text chat (individual and group), in-app weekly monitoring and reminders will be available to participants for up to 12 months after completing the study. The dietitian/coach support is flexible and can be tailored to individual participants, based on their needs.

### Control group

The control group will receive standard care and dietary/exercise advice which will follow the National Health Service (NHS), National Institute for Health and Care Excellence and the European Society of Cardiology guidelines^18 26 63^ for promoting low-moderate intensity physical activity. BP and lipid management will adhere to clinical guidelines, with statin therapy adjusted based on CV risk.^63^ Health coaching advice will be provided to participants at baseline and follow-up visits.

### Study outcomes

Study endpoints are summarised in table 2 and discussed in brief below.

**Table 2:** Summary or key trial objectives and endpoints

Primary objective	To investigate whether weight loss achieved with a low-energy MRP in obese adults with HF and preserved ejection fraction improves physical function (as determined by age, sex-adjusted Six-Minute Walk Test (6MWT) distance) compared with standard clinical care with attention control.
Secondary objectives	To examine the effect of MRP-mediated weight loss on: Reverse cardiovascular remodelling assessed at 12 weeks and 24 weeks. Improvement exercise capacity and preservation of muscle power. Improvement in HF symptoms and quality of life. Improvement in skeletal and muscle energetics. Improvement in metabolic profile. Physical activity as determined by accelerometery.
Primary end point	The change in distance walked during 6MWT between baseline and 12 weeks.
Primary imaging end point	Change in the left atrial volume indexed to height—between baseline and 12 weeks
Key secondary endpoints	Key changes in indices between baseline and 12 weeks: HFpEF symptoms: Kansas City Cardiomyopathy Questionnaire, Borg dyspnoea scale during exertion, SARC-F and Edmonton frailty scale. Body composition and adiposity including visceral and body adipose tissue volumes (determined on MRI). Key imaging endpoints include change in LV mass: volume, LV mass, LV mass/height, atrial volumes, systolic strain and diastolic strain rates, myocardial blood flow/perfusion reserve, extracellular volume, global myocardial perfusion reserve as a marker of microvascular function, circumferential and longitudinal PEDSR and myocardial systolic strain/strain rates (circumferential and longitudinal), arterial stiffness (mean ascending aortic distensibility). Echocardiographic mitral inflow velocity (E), myocardial relaxation (E’) and E/E’, a non-invasive marker of LV filling pressure. Diastolic dysfunction will be graded. Skeletal muscle strength. Levels of circulating fibroinflammatory biomarkers at baseline and postintervention. Daily physical activity behaviours as measured by accelerometery.
Exploratory	Qualitative data from semistructured interview. Skeletal and cardiac energetics assessed with 31P-MR spectroscopy. Proteomic, metabolomic and DNA samples analyses subject to additional funding.

HFheart failureHFpEFheart failure with preserved ejection fractionLVleft ventricularMRPmeal-replacement planSARC-FStrength, Assistance with walking, Rise from a chair, Climb stairs and Falls

#### Primary outcome

The trial’s primary outcome is the change in the distance traversed during the 6MWT (in metres) between baseline and 12 weeks.

The primary imaging outcome is the absolute change in the CMR-derived LAVi to height between baseline and 12 weeks.

#### Key secondary outcomes

The complete list of secondary outcomes is summarised in table 2. In summary, the key secondary outcomes will assess change between baseline and 12 weeks in the following:

- Changes in body composition: change in body composition, visceral adiposity and body weight.
- Change in CV risk profile: change in BP measured with sphygmomanometer. Change in fasting glucose and glycaemic control (HbA1c) where applicable, lipid profile, markers of myocardial stress (troponin I and NT-proBNP levels). These outcomes will be measured with blood tests.
- Change in severity of symptoms of HFpEF will be assessed with a difference in KCCQ score.
- Change in markers of frailty will be assessed with a difference in EFS score, SARC F score.
- Change in sarcopenia measures will be assessed by measuring the absolute difference in the maximum handgrip strength test and MRI-derived SMI.

#### Key cardiac imaging secondary outcomes

- Change in CMR-derived indices of LV remodelling (LV mass: volume ratio) and CMR-derived LV strain between baseline and 12 weeks.
- Change in CMR-derived MPR between baseline and 12 weeks.
- Change in echocardiogram derived E/e’ (measure of diastolic function) between baseline and 12 weeks.

#### Exploratory outcomes

Exploratory outcomes will be centred around the data from the optional qualitative interview, musculoskeletal and spectroscopy substudies.

- Qualitative data from semistructured interview.
- Skeletal and cardiac energetics assessed with 31P-MRS.
- Proteomic, metabolomic and DNA analyses from blood samples (subject to additional funding).
- Skeletal muscle biopsy analysis for muscle fibre type and distribution, fat infiltration and inflammatory changes.

### Study reporting

On completion of the study, a Consolidated Standards of Reporting Trials diagram will be produced, which will include the number of patients: approached, consented, meeting eligibility criteria, reasons for exclusion, randomised and completing the study.

## Statistical methods and analysis

### Sample size calculation

The power calculation for 6MWT is based on an observational HFpEF study from our group where mean distance on 6MWT was 180±91.7 m.^64^ To detect a clinically meaningful difference of 55 m^65^,^67^ between MRP and control group, requires 88 subjects to complete the study (80% power at 0.05 significance level). Allowing for an overall drop-out of 20% (assuming up to 30% drop-out in the MRP arm and <10% drop-out in standard care), 110 participants will be recruited.

The CMR is powered to detect a 10 mL/m^2^ reduction in LAVi to body surface area and requires 44 participants per group. Additionally, detecting an improvement in MPR from 1.74±0.76 to 2.2±0.76, based on our pilot data, requires 42 participants per group. Therefore, a group of 110 will maintain >80% power (2-sided alpha, p<0.05) for LA volume indexed (allowing 20% drop out) and MPR (allowing 25% drop out).

### Statistical analysis

The distribution of data will be assessed for normality using histograms, the Shapiro-Wilk test and Q-Q plots. Descriptive statistics will be used to characterise the groups comprising only those participants who have completed the trial, as per the per-protocol analysis criteria. Continuous data will be expressed as mean (±SD), if normally distributed or median (IQR 25%–75%) if not. Categorical variables will be reported as count and percentage. For the analysis of the primary outcome, the MRP group will be compared with the control group, using linear regression adjusted for stratification factors (sex, ethnicity and baseline SGLT2-i use) and baseline distance walked during 6MWT. The treatment effect will be presented as a point estimate, 95% confidence intervals and p value. Changes in the key secondary outcomes will also be assessed using linear regression with the same stratification factors as the primary outcome.

Additionally, the relationships between clinical and imaging markers (cardiac function, adiposity and skeletal myopathy) at baseline and their changes will be correlated with exercise capacity and changes therein among completers. Multivariate regression analyses will be employed to discern significant independent predictors of outcomes. To ensure methodological rigour, a comprehensive statistical analysis plan will be developed and formally approved by the study statistician before the commencement of these analyses.

### PPI statement

Our trial design incorporated PPI inputs from four representatives of patients with HFpEF, heart failure with reduced ejection fraction and T2D. The 6MWT was selected as the primary endpoint, following PPI feedback prioritising tangible treatment benefits, such as enhanced walking ability. PPI representatives also contributed to developing qualitative questionnaire questions (topic guide is presented in online supplemental file: appendix 3). We pledge ongoing PPI engagement, and we have appointed one PPI member to our Trial Steering Committee (TSC). We will share the trial outcomes with our PPI heart failure group on completion.

### Trial oversight and governance

The study sponsor is University of Leicester. A Data Monitoring Committee was not deemed necessary for this open-label RCT given the intervention is already recommended in primary care and commercially available for independent purchase. Instead, oversight will be maintained through the TSC, which will review safety data and monitor trial conduct, and have the authority to recommend trial modification or termination based on predefined stopping criteria. All safety data will be systematically collected throughout the trial using standardised case report forms, and all AEs, including serious AEs, will be reported promptly to the TSC and relevant regulatory bodies in accordance to the sponsors standard operating procedures (SOPs). Study audit will be conducted by the sponsor.

### Current status and timescale

Recruitment started in December 2023 and the follow-up of the last participant is expected to complete by the end of 2025. At the time of submission, 24 participants have been randomised.

## Discussion

The burden of obesity-related HFpEF is increasing, and treatment options are limited. Obesity is emerging as one of the major modifiable risk factors responsible for HF development.^68^ Weight loss is the traditional treatment for obesity, and it has been shown to confer favourable CV and risk profile improvements in those with obesity, and those with obesity and T2D even in the absence of HF.^69^,^71^ The AMEND Preserved trial benefits from randomised controlled design and comprehensive multiparametric phenotyping of participants. The AMEND Preserved trial explores the impact of weight loss through low-calorie MRP on cardiac and systemic manifestations of HFpEF, with a particular focus on under-represented groups. This trial represents a pivotal step towards understanding the multifaceted nature of HFpEF and developing more effective treatments which produce notable benefits to patients. If successful, the pragmatic design of the AMEND Preserved trial combined with the commercial availability of the MRP will make it directly translatable to the large cohort of patients struggling with weight management within the heart failure services. The use of MRP is currently available on the UK NHS prescription in those living with obesity and T2D but not heart failure. The results of this trial would potentially open another treatment avenue for the weight management services which are currently limited in options for treating patients living with obesity and HFpEF.

To date, three large randomised controlled trials of weight loss interventions in obesity-related HFpEF with low-calorie MRP or use of semaglutide in those with obesity-related HFpEF (STEP HFpEF)^19 21^ and those with obesity and T2D-related HFpEF (STEP-HFPEF DM).^20^ Although these studies were conducted nearly a decade apart and differed in methodology as well as presence or absence of T2D, the results support the notion that weight loss is conducive to improvements in exercise capacity, improvement in quality of life and reduction of CV risk profile.^19 21^ Nonetheless, the studies exhibited limitations in participant phenotyping, offering very limited insights into cardiac structure and function beyond basic echocardiographic or MRI-based volumetric measurements.^19 21^ Additionally, the evaluation of inflammatory markers was constrained, with only CRP levels assessed in STEP-HFpEF and CRP and interleukin-6 levels only assessed in the 2×2 study.^21^ The examination of sarcopenia, alterations in body composition in relation to symptoms, cardiac indices and the muscle-to-fat ratio was notably absent, precluding a thorough understanding of weight loss’s impact on these important areas and the true pathophysiological relationship with HFpEF symptoms. The AMEND preserved trial aims to bridge this gap by incorporating comprehensive multiparametric phenotyping of multiple organs at both baseline and follow-up stages, thereby enriching the understanding of these dynamics in the HFpEF population.

There are a number of potential limitations of the AMEND Preserved trial. First, the sample size determination primarily focused on clinically significant improvements in the 6MWT, which potentially limits the evaluation of secondary endpoints such as improvement in KCCQ scores, body composition and strength and skeletal muscle mass. Although, in the STEP trials, there was a small difference in the 6MWT traversed between those with T2D and those without, we chose not to stratify by the presence of diabetes at baseline because the use of SGLT2-i might be a more relevant factor in influencing outcomes in individuals with HFpEF. Since SGLT2-inhibitors have demonstrated benefits independent of glycaemic control, stratifying by their use could capture this therapeutic effect more directly, regardless of diabetic status.

Exercise-based intervention, although beneficial for CV fitness, was not included in the AMEND-Preserved design due to challenges posed by obesity and limited exercise tolerance. We did consider a design which also incorporated exercise training for preservation of lean muscle mass, however, our PPI group was not supportive of such a design. This is further supported by a recent network meta-analysis from our group comparing the efficacy of lifestyle-based interventions (diet vs exercise) on improving exercise tolerance and quality of life in patients with HFpEF.^72^ The analysis concluded that high-intensity interval training was the most efficient way of improving exercise tolerance in those with HFpEF and normal body weight. In those with HFpEF who were overweight or obese, the exercise tolerance only improved with addition of weight loss via hypocaloric diet.^72^ This finding highlights the important message that weight loss is an essential step to improving exercise tolerance in those living with HFpEF and elevated body weight. Weight loss interventions may likely be followed by exercise-based therapies once the weight is reduced and exercise tolerance is improved and could be tested in future trials.

If successful, the results of the AMEND-Preserved trial may pave the way for a large phase 3 clinical trial to investigate clinical outcomes and patient-reported outcome measures.

## Ethics and dissemination

The study is conducted in accordance with the principles of the 1996 Declaration of Helsinki, International Conference on Harmonisation-Good Clinical Practice (ICH-GCP) guidelines. The trial has ethical approval from the national research ethics service, the East Midlands—Derby Research Ethics Committee (22/EM/0215). Any modifications affecting the study’s conduct will require a formal protocol amendment, approved by the trial sponsor and, if necessary, the Research Ethics Committee (REC) prior to implementation. Approved amendments will be communicated to all recruitment sites. The final dataset will be accessible to the PIs and the chief investigator. As the trial sponsor, the University of Leicester holds indemnity insurance for compensation purposes, should the need arise.

It is anticipated that data collection will be completed by end of April 2025, and the study results will be submitted for publication within 6 months of completion. Participants who opted in to be informed of the study outcomes will receive a lay summary of key results on trial completion. Authorship of trial outcome publications will follow ICMJE authorship guidelines.

## supplementary material

10.1136/bmjopen-2024-094722 online supplemental file 1
