# Supplementary material for: The Protocol for the Multi-Ethnic, multi-centre raNdomised controlled trial of a low-energy Diet for improving functional status in heart failure with Preserved ejection fraction (AMEND Preserved)
Source: BMJ Open. 2025 Jan 28;15(1):e094722. doi: 10.1136/bmjopen-2024-094722 (PMC11781100; doi:10.1136/bmjopen-2024-094722)
Supplement: online supplemental file 1 [file bmjopen-15-1-s001.docx]

## Appendix 1: Diuretic and antihypertensive drug adjustment algorithm for the AMEND Preserved trial.

It is recognised that most patients will be on a combination of diuretic(s) and antihypertensives.  These medications will be reduced or stopped to reduce the risk of hypotension, as the MRP leads to marked reductions in blood pressure which is apparent within the first few days. During the study, participants weight and BP will be monitored by the participant. Medication and symptoms will be reviewed fortnightly for first 4 weeks, then every 4 weeks and as required in response to worsening symptoms reported by patients.  Medication will be reintroduced or reduced as necessary depending on increasing breathlessness, fluid retention, weight gain and or blood pressure response.

**The Algorithm for adjustment of cardiac medications in the AMEND study participants**

For those on polypharmacy, at baseline visit the recommended order of withdrawal will be: first line will be to adjust calcium channel blockers (adjust or halve dose as per table 1). Adjust or stop beta blockers as second line (unless patient is in atrial fibrillation), adjust ACEi/ARB (depending on systolic blood pressure (SBP) and number of agents being reduced (steps 1-2)). Finally, as patients with HFpEF are prone to fluid retention, diuretic doses will be adjusted only if necessary (symptomatic hypotension despite cessation of anti-hypertensives) and cautiously after careful review of clinical history. In general, a cautious approach of withdrawal of one agent at a time is recommended but in individual cases multiple drug adjustments can be made in one visit based on clinician’s judgement. Depending on which medications the patient takes, the clinician can start at the relevant step as outlined below, and move through subsequent steps depending on patent’s haemodyamics as subsequent visit. The proposed algorithm below is a guide, and should be used with clinical judgement and individualised to each patient based on clinical history and symptoms.


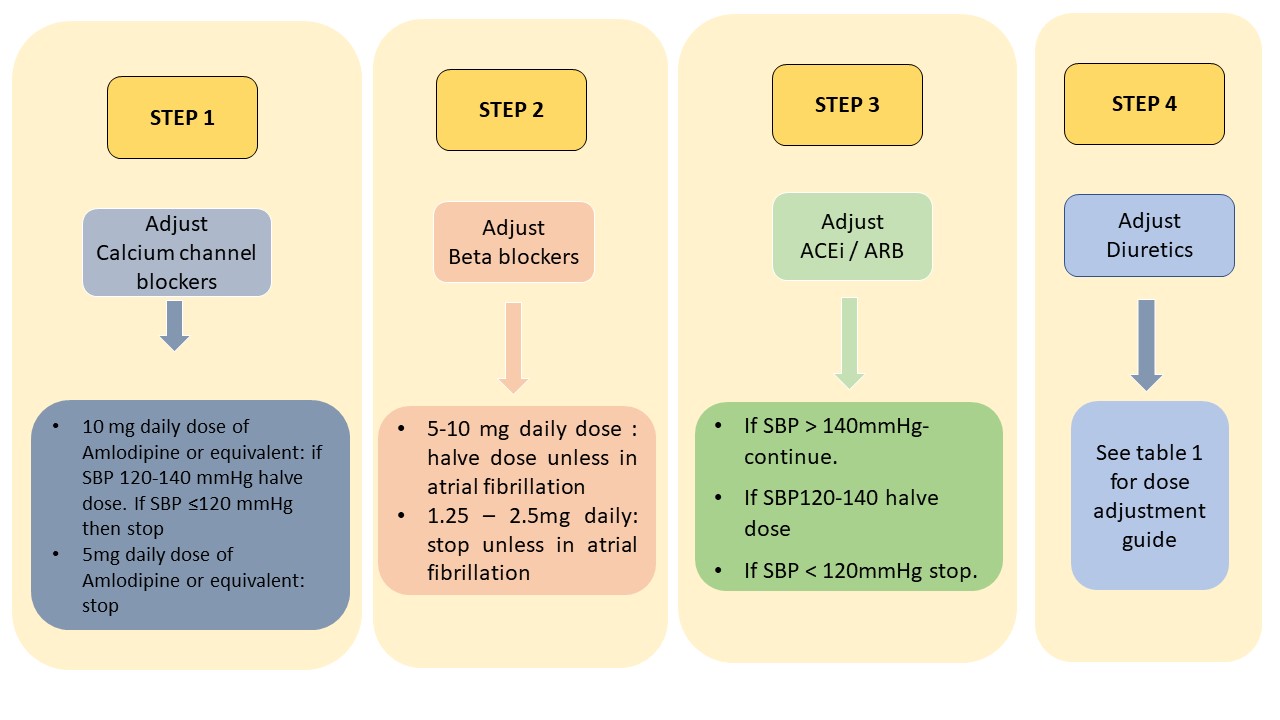


**Appendix 1, table 1. Recommended adjustment to diuretic doses if diuretic adjustment is required.**

**Appendix 2. Decision algorithm for the management of glucose lowering therapy in adults living with type 2 diabetes mellitus on injectable glucose lowering therapies (with and without oral glucose lowering therapy)**

**For patients on single or multiple therapy – please follow the table below**

| **Group** | **Examples include** | **Recommendation** |
| --- | --- | --- |
| **Premix insulin** | NovoMix, HumulinM3, Humalog Mix, Mixtard | Swap to basal only and follow advice below |
| **Basal insulin** | Degludec, Glargine, Detemir, Insulatard, Humulin I, Insuman Basal, Toujeo (Glargine U300) | Total daily does:  < 30 IU/d stop basal  ≥ 30 IU/d Reduce basal by 50% |
| **Basal/bolus** | Short acting: actrapid, HumulinS  Rapid acting: Novorapid, Humalog, Apidra | Total daily dose  < 30 IU/d stop basal  ≥ 30 IU/d Reduce basal by 50%  Reduce prandial/bolus insulin dose by 50% |
| **GLP-1** | Dulaglutide, exenatide, liradlutide, lixisenatide, semaglutide | Continue GLP-1 |
| **Insulin and GLP-1 combined** | Combinations of above | Follow advice for basal insulin above and continue GLP-1 |
| **SGLT2 inhibitors** | Dapagliflozin, empagliflozin, canagliflozin | Continue SGLT2i and offer ketone strips for testing |
| **SGLT2inhibitoris plus insulin** | Combinations of above | Continue SGLT2i  Cautious reduction in insulin in those with HbA1c > 8 at baseline |

**Troubleshooting events of hyperglycaemia during MRP in individuals on insulin therapy**

- Patient should self-monitor pre-breakfast blood glucose (OR continue current regimen if testing is more frequent).
- If fasting glucose > 7 mmol/L for 7 days of > 10 mmol for 3 consecutive days, check for episodic illness or alternative causes before restating drugs in the below order
- Continue SGLT2inhibitor

| **Order of reintroduction of drugs** | |
| --- | --- |
| 1 | Metformin |
| 2 | Add GLP-1 |
| 3 | Add DPP-4i not if on GLP-1 |
| 4 | Add sulfonylurea, Glitazone |
| 5 | If remained on basal insulin, follow EASD and ADA guidance for intensification e.g increase 2 IU every 3 days until target blood glucose is achieved. |

**3. Decision algorithm for the management of glucose lowering therapy in adults living with type 2 diabetes mellitus on ORAL glucose lowering therapies only**

| **Group** | **Examples include** | **Recommendation** |
| --- | --- | --- |
| Metformin |  | Stop (if slow release, stop 72 hours prior to commencing MRP) |
| SGLT2-inhibitor | Dapagliflozin, empagliflozin, canagliflozin | Continue SGLT2i and offer ketone strips for testing |
| Sulphyonlyurea | Gliclazine. Glibenclamide, glimepride, tolbutamide | STOP |
| Glitazone |  | STOP |
| Glinide |  | STOP |
| DPP-4i | Alogliptin, linagliptin, sitagliptin | STOP |
| Acarbose |  | STOP |

**3.1 Troubleshooting events of hyperglycaemia during MRP in individuals on oral hypoglycaemics**

- Patient should self-monitor pre-breakfast blood glucose (OR continue current regimen if testing is more frequent).
- If fasting glucose > 7 mmol/L for 7 days of > 10 mmol for 3 consecutive days, check for episodic illness or alternative causes before restating drugs in the below order

| **Order of reintroduction of drugs** | |
| --- | --- |
| 1 | Metformin |
| 2 | Add GLP-1 |
| 3 | Add DPP-4i but not if on GLP-1 |
| 4 | Add sulfonylurea, Glitazone or basal insulin |

**Appendix 3. Qualitative interview topic guide**

**AMEND preserved**

**Interview Topic Guide**

1. **Welcome and introduction**
2. **Seek consent to continue and to audio-record the interview (if applicable).**
3. **Let them know that no personal identifiable data will be recorded and a participant number will be allocated to them**
4. **Utilising the question prompts on the next page, conduct the interview.**
5. **At the end of the interview, ask the participant regarding anything they think we have not covered? Example question:** Is there anything that we haven’t covered in the interview that you think we should know or think about?
6. **Check that they participant is well and there are no safety concerns.**
7. **Closing and thanks -** check that the participant is still happy for you to use all the information provided and offer the possibility to erase sections of the recording.
8. Thank the participant for their time and contribution.

| **Theme** | **Main question** | **Supporting questions** |
| --- | --- | --- |
| **Short interview on first visit** | | |
| **Participant management of heart failure (HF)** | Tell me about your HF |  |
|  | How do you cope in daily life with self-management of the disease? | How does it affect your work, social life, family life? |
| **Lifestyle management** | Do you sometimes feel it is hard to do all things to take care of your health? | How much time do you spend on exercise, and diet based on advice from health professionals |
| **Previous attempts to change lifestyle** | Have you ever made attempts to lose weight through diet? | How did it go? What made you relapse? |
| **Short interview at last visit** | | |
| **Management of HF during trial** | How did you cope in daily life with self-management of the diet during the trial? | What new skills have you learnt to take care of yourself |
| **Lifestyle management** | How did the diet affect your day-to-day management of your HF | How much time did you spend on diet based on advice from health professionals |
|  |  | How did that affect your lifestyle (your family life) |
| **Challenges in adherence during trial** | What were the main challenges of diet? |  |
|  | Do you ever skip any of the things you should have done or done things you should not have in terms of food intake during the 12 weeks? | Do you perceive that you follow the advice you get?  If yes, what has been the most helpful advice?  If no, why? |
| **Sustained change or relapse** | What made you relapse?  What made you carry on? | Is there something you did to:   1. Keep your spirits up 2. Keep your motivation going |
| **Status since the intervention stopped** | Do you find that your diet / eating habits have changed as a result of being in the trial? |  |
| **Reflections about adherence** | What do you find the hardest about sticking to the diet? | What could be done better to help you adhere |
| **Support and level of importance** | How important is healthy eating to you? |  |
| **Proposal on how to increase adherence to lifestyle in lifestyle interventions** | Do you have any suggestions as to how we could support you better in maintaining the diet during the trial? |  |
| **Ending** | Is there anything you wish to add |  |

Interview prompts
