# Supplementary material for: The Protocol for the Multi-Ethnic, multi-centre raNdomised controlled trial of a low-energy Diet for improving functional status in heart failure with Preserved ejection fraction (AMEND Preserved)
Source: BMJ Open. 2025 Jan 28;15(1):e094722. doi: 10.1136/bmjopen-2024-094722 (PMC11781100; doi:10.1136/bmjopen-2024-094722)
Supplement: Supplementary file 2 [file bmjopen-2024-094722.reviewer_comments.pdf]

PEER REVIEW HISTORY

BMJ Open publishes all reviews undertaken for accepted manuscripts. Reviewers are asked to complete a checklist review form and are provided with free text boxes to elaborate on their assessment. These free text comments are reproduced below.

ARTICLE DETAILS

Title (Provisional)

Protocol for the Multi-Ethnic, multi-centre raNdomised, controlled trial of a low-energy Diet for improving functional status in Heart failure with Preserved ejection fraction (AMEND-Preserved)

Authors

Bilak, Joanna M; Squire, Iain; Wormleighton, Joanne V; Brown, Rachel L; Hadjiconstantinou, Michelle; Robertson, Noelle; Davies, Melanie J; Yates, Thomas; Asad, Mehak; Levelt, Eylem; Pan, Jiliu; Rider, Oliver; Soltani, Fardad; Miller, Christopher; Gulsin, Gaurav Singh; Brady, Emer M; McCann, Gerry P

This article was not externally reviewed at BMJ Open. Protocols that have been independently assessed prior to submission to BMJ Open are usually fast-tracked to publication on the grounds that further substantial changes will not be possible. This independent assessment will usually be external, independent review for both a substantial grant award from a non-commercial or government funder and ethics approval.
